# Comparison of clinical hysterectomy indications with ai-based recommendations: a prospective study

**DOI:** 10.1038/s41598-025-19216-y

**Published:** 2025-10-09

**Authors:** Saltuk Buğra Arıkan, Can Dinç, Mustafa Özer, Ömer Faruk Öz, M. Ilkin Yeral

**Affiliations:** Department of Gynecology and Obstetrics, Akdeniz University, Antalya, 07010 Turkey

**Keywords:** Artificial intelligence, Abnormal uterine bleeding, Hysterectomy, Gynecology, Clinical decision support system, Machine learning, Urogenital reproductive disorders, Physical examination, Reproductive signs and symptoms, Computational models, Computational science

## Abstract

This prospective study evaluated ChatGPT-4 as a decision-support tool by comparing its treatment recommendations with clinical decisions for 87 women (aged 40–65 years) scheduled for hysterectomy. Demographic, clinical, and ultrasonographic data were standardized and submitted to the GPT-4 model, which generated evidence-based treatment suggestions. ChatGPT-4 recommended hysterectomy in 70.1% of cases, aligning with the original clinical decision, and suggested alternatives such as myomectomy (10.3%), hysteroscopy (8.0%), or medical therapy (4.6%) in others. These alternative options were retrospectively judged as appropriate in selected scenarios. Although the model demonstrated guideline-consistent reasoning, it lacked access to imaging, laboratory results, and physical examination findings. The study’s single-center design, absence of sample size calculation, and purely descriptive nature further limit generalizability. Large language models may complement clinical decision-making but should not replace physician expertise. Multicenter studies are needed to validate their reliability and clinical applicability.

**Supplementary Information:**

The online version contains supplementary material available at 10.1038/s41598-025-19216-y.

## Introduction

Artificial intelligence (AI) is increasingly integrated into medical practice, offering potential to enhance diagnostic precision, optimize treatment strategies, and improve patient outcomes^1,2^. In surgical decision-making, large language models (LLMs) such as ChatGPT-4 have shown performance comparable to experienced clinicians^3,4^. While AI has been applied in surgical planning, intraoperative guidance, and postoperative care across various specialties, its role in gynecologic surgery decision-making remains largely unexplored^5–7^.

Hysterectomy is among the most common gynecologic procedures, performed for both benign and malignant conditions^8^. As clinical care becomes more complex and data-driven, AI systems have the capacity to identify patterns, synthesize evidence, and support treatment planning. Unlike conventional decision-support tools, LLMs can adapt to new inputs and integrate emerging medical knowledge in real time^9,10^.

This study evaluates ChatGPT-4’s ability to recommend appropriate treatment options for patients scheduled for hysterectomy at the Department of Obstetrics and Gynecology, Akdeniz University. Standardized patient scenarios incorporating demographic, clinical, and ultrasonographic data were used to generate AI-based recommendations. The primary objective was to compare these with actual clinical decisions to assess concordance, effectiveness, and limitations, and to discuss the implications of integrating LLMs into gynecologic practice for responsible and evidence-based adoption^11^.

## Materials and methods

Between June 1 and November 1, 2023, a total of 87 patients aged 40 to 65 years who were scheduled for hysterectomy at the Department of Obstetrics and Gynecology, Akdeniz University, were included in this prospective observational study. Comprehensive clinical data, including patient histories, demographic information, prior treatments, presenting complaints, and ultrasound findings, were systematically collected by the research team. All patient data were fully anonymized before analysis to ensure privacy and confidentiality in accordance with institutional and international guidelines.

Standardized clinical scenarios were entered into the GPT-4 Turbo large language model (OpenAI, San Francisco, CA, USA) via the ChatGPT Plus interface. No Research Resource Identifier (RRID) or DOI is currently available for this proprietary model. For each case, the model was asked to interpret the clinical information and recommend the most appropriate treatment option, providing a literature-based rationale. The AI model was explicitly informed that the clinical team had planned hysterectomy for each patient and was asked whether it agreed with this decision or suggested an alternative, again with supporting rationale.

Statistical analyses were performed using IBM SPSS Statistics for Windows, Version 26.0 (IBM Corp., Armonk, NY, USA). The Shapiro–Wilk test was applied to assess normality, and categorical variables were compared using Chi-square tests. A p-value of < 0.05 was considered statistically significant.

This study was approved by the Akdeniz University Faculty of Medicine Clinical Research Ethics Committee on May 10, 2023 (Approval No: KAEK-390).

### Allocation and sample size estimation

This study was designed as a prospective observational analysis including all eligible patients who underwent hysterectomy between June 1 and November 1, 2023, at the Department of Obstetrics and Gynecology, Akdeniz University. Patient enrollment was based on consecutive admissions during the specified study period. As the study aimed to include the complete population meeting the inclusion criteria within this timeframe, no a priori sample size calculation was performed. The final sample size (*n* = 87) reflects all eligible patients treated during the study period. While this approach ensures comprehensive coverage of the target population, it may limit the statistical power to detect smaller effect sizes.

## Procedures

In this study, we utilized the GPT-4 Turbo large language model (OpenAI, San Francisco, CA, USA), the fourth-generation version of the Generative Pretrained Transformer (GPT) series. It was trained on large-scale textual datasets, including scientific literature, using deep learning techniques. Its advanced natural language processing capabilities allow it to interpret complex clinical narratives and generate contextually appropriate responses. For each patient, a standardized and anonymized clinical scenario was constructed using detailed and structured input variables to ensure reproducibility. These included age, parity, mode of delivery, menopausal status, presenting symptoms, comorbid conditions, prior interventions, current medications, family history of malignancy, ultrasonographic findings (e.g., fibroid size and type, endometrial thickness), relevant laboratory data, and any available pathology reports. Each scenario was entered into the GPT-4 Turbo model via the ChatGPT Plus interface. The model was presented with multiple potential treatment options including hysterectomy, myomectomy, hysteroscopy, medical therapy, levonorgestrel-releasing intrauterine device (LNG-IUD), and staged procedures such as hysteroscopy followed by hysterectomy. For each case, it was tasked with selecting the most appropriate management strategy and providing a justification based on current clinical guidelines and peer-reviewed literature.

### Statistical analysis

Data analysis was performed using IBM SPSS Statistics for Windows, Version 23.0 (IBM Corp., Armonk, NY, USA). Continuous variables were expressed as mean ± standard deviation (SD), while categorical variables were summarized as percentages (%). The Shapiro–Wilk test was applied to assess the distribution of continuous data, which did not meet the criteria for normality. Categorical comparisons were performed using Pearson’s Chi-square test and, when appropriate, Pearson’s Exact Chi-square test. A p-value of < 0.05 was considered statistically significant.

## Results

In this study, the demographic characteristics, clinical histories, and surgical outcomes of 87 women who underwent hysterectomy were analyzed. The key demographic and clinical parameters of all patients are presented in Table 1.

**Table 1:** Demographic and clinical characteristics of the study population (*n* = 87).

Variable	Category	*N* (%)	Mean ± SD
**Age**			48.7 ± 7.5
**Gravida**	1	10 (11.5)	
	2	37 (42.5)	
	3	23 (26.4)	
	4	8 (9.2)	
	≥ 5	6 (6.9)	
**Mode of Delivery**	Vaginal Delivery	51 (58.6)	
	Cesarean Section	16 (18.4)	
	Vaginal Delivery& Cesarean Section	17 (19.5)	
**Menopausal Status**	Menopausal	20 (23.0)	
	Premenopausal	67 (77.0)	

The mean age was 48.7 ± 7.5 years. A history of two pregnancies was most common (42.5%), and 58.6% had delivered vaginally. At the time of evaluation, 77.0% were premenopausal.Patients’ chief complaints and prior treatments are shown in Table 2.

**Table 2:** Chief complaints and prior medical treatments of the study population (*n* = 87).

Variable	Category	Mean ± SD *N* (%)
**Current Chief Complaint**	IMB	9 (10.3)
	HMB	24 (27.6)
	IMB&HMB	8 (9.2)
	Irregular Menstruation	27 (31.0)
	PMB	18 (20.7)
**History of medical treatment for current complaint**	Yes	41 (47.1)
	No	46 (52.9)
**If yes**,** type of medical treatment**	COC	18 (20.7)
	Hormonal therapy	22 (25.3)
	LNG-IUD	11 (13.8)
	No	47 (54.0)

Combined Oral Contraceptive (COC), Diabetes Mellitus (DM), Heavy Menstrual Bleeding (HMB), Hypertension (HT), Intermenstrual Bleeding (IMB), Levonorgestrel-Releasing Intrauterine Device (LNG-IUD), Polycystic Ovary Syndrome (PCOS), Postmenopausal Bleeding (PMB).

The most common complaints were irregular menstruation (31.0%) and heavy menstrual bleeding (27.6%), followed by postmenopausal bleeding (20.7%), intermenstrual bleeding (10.3%), and combined IMB and HMB (9.2%). Nearly half (47.1%) had received prior medical therapy, most often hormonal therapy (25.3%), COCs (20.7%), or LNG-IUD insertion (13.8%).

Table 3 summarizes physical examination and Transvaginal Ultrasound (TVUS) findings.

**Table 3:** Physical examination and pelvic ultrasonography findings of the study population (*n* = 87).

Variable	Category	*N* (%)
**TV-USG findings related to the endometrium**	Irregular Endometrium	18 (20.7)
	No Endometrial Hyperplasia	38 (43.7)
	Endometrial Hyperplasia Present	27 (31.1)
	Suspicious Mass	4 (4.6)
**Presence of uterine myoma**	Yes	61 (70.1)
	No	26 (29.9)
**Type of myoma (FIGO classification)**	Type 0–2	7 (8.0)
	Type 3–5	35(40.1)
	Type 6–7	14 (16)
	Adenomyozis	5 (5.7)
**Myoma size**	< 3	2 (2.3)
	3–5 cm	16 (18.4)
	5–10 cm	15 (17.2)
	> 10	16 (18.4)
	Multiple intramural myoma	12 (13.8)
**Polyp**	Yes	14 (16.1)
	No	72 (82.8)
**Low hemoglobin**	Yes	30 (34.5)
	No	57 (65.5)

*TV-USG*,* Transvaginal Ultrasonography; FIGO*,* International Federation of Gynecology and Obstetrics.*

TVUS revealed no endometrial hyperplasia in 43.7% of patients, while 31.1% had hyperplasia, 20.7% showed irregular endometrium, and 4.6% had a suspicious mass. Uterine myomas were present in 70.1%, most often type 3–5 (40.1%). Lesions ≥ 10 cm were most frequent (18.4%), followed by 3–5 cm (18.4%) and 5–10 cm (17.2%). Adenomyosis was detected in 5.7%, multiple intramural myomas in 13.8%, and small myomas (< 3 cm) in 2.3%. Endometrial polyps were suspected in 16.1%, and anemia was present in 34.5%.

Table 4 summarizes endometrial biopsy findings and AI-generated treatment recommendations.

**Table 4:** Endometrial biopsy findings and AI-generated treatment recommendations for the study population (*n* = 87).

Variable	Category	*n* (%) *N* (%)
**Endometrial biopsy findings**	Normal	58 (66.7)
	Endometrial Hyperplasia without atypia	11 (12.6)
	EIN (+)	4 (4.6)
	Polyp	11 (12.6)
	Malignancy	3 (3.4)
**Treatments performed**	Hysterectomy	87 (100.0)
	Myomectomy	N/A
	Hysteroscopy	N/A
	Medical Treatment	N/A
	LNG-IUD	N/A
	Follow-up	N/A
	Hysterectomy after hysteroscopy	N/A
**Treatment options recommended by AI**	Hysterectomy	61 (70.1)
	Myomectomy	9 (10.3)
	Hysteroscopy	7 (8.0)
	Medical Treatment	4 (4.6)
	LNG-IUD	2 (2.3)
	Follow-up	1 (1,1)
	Hysterectomy after hysteroscopy	3 (3.4)

*EIN*,* Endometrial Intraepithelial Neoplasia; LNG-IUD*,* Levonorgestrel-Releasing Intrauterine Device.*

Pathological evaluation after endometrial biopsy showed normal histology in 58 patients (66.7%), hyperplasia without atypia in 11 (12.6%), polyps in 11 (12.6%), endometrial intraepithelial neoplasia (EIN) in 4 (4.6%), and malignancy in 3 (3.4%).

From 87 standardized clinical scenarios assessed by AI, hysterectomy was recommended for 61 patients (70.1%). Less common suggestions included myomectomy (9, 10.3%), hysteroscopy (7, 8.0%), medical therapy (4, 4.6%), hysterectomy after hysteroscopy (3, 3.4%), LNG-IUD (2, 2.3%), and follow-up (1, 1.1%).

**Fig. 1:**
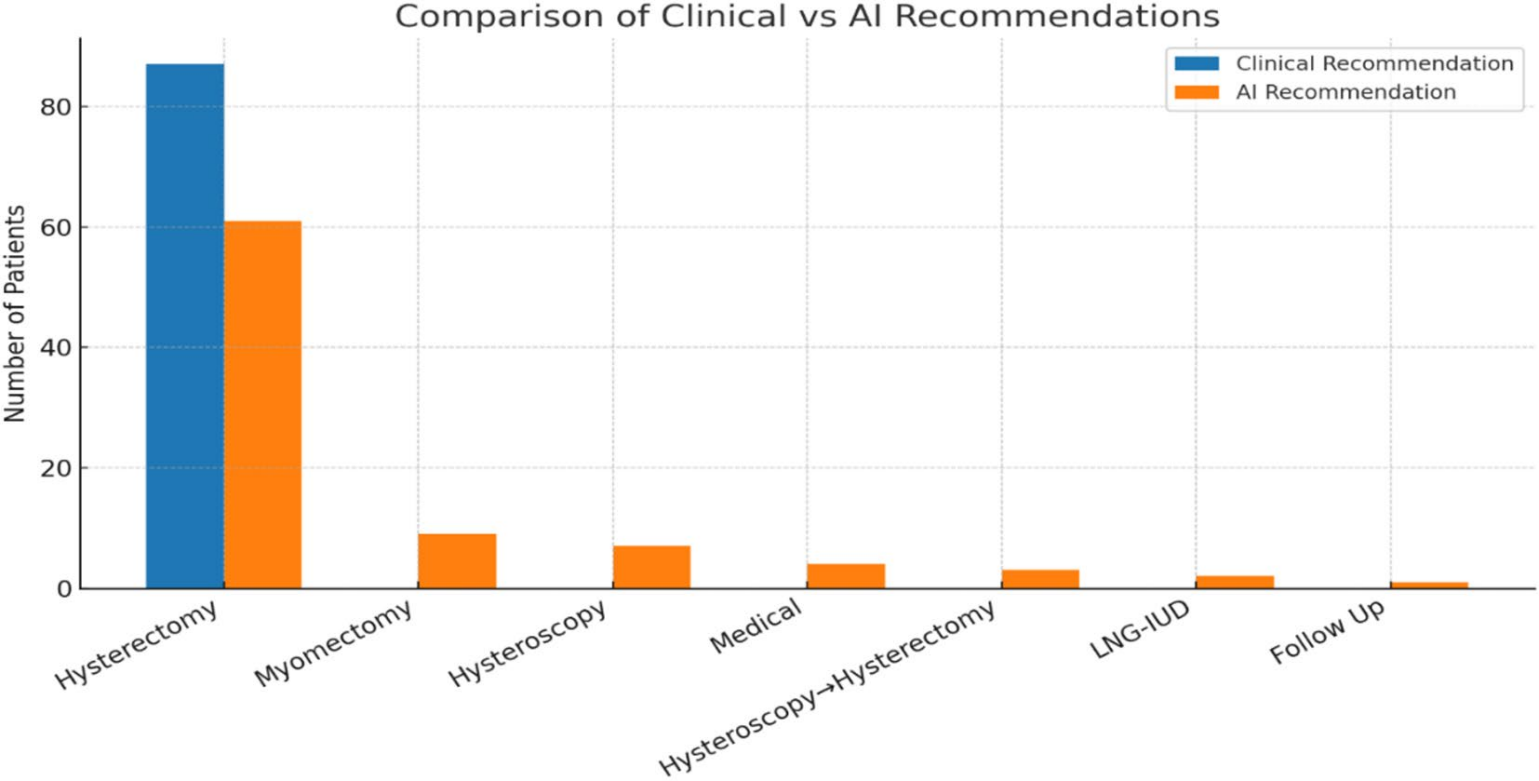
Comparison of clinical recommendations and AI-generated treatment suggestions for 87 patients initially scheduled for hysterectomy.

As shown in Fig. 1, each case scenario incorporating clinical presentation, ultrasonographic findings, and relevant history was entered into the ChatGPT-4 model using a standardized prompt. The bar chart illustrates concordance and divergence between AI-generated and clinical decisions, along with the distribution of alternative strategies such as myomectomy, hysteroscopy, medical therapy, LNG-IUD insertion, and follow-up.

## Discussion

This prospective study assessed the concordance between clinical indications for hysterectomy at our institution and treatment recommendations generated by ChatGPT-4 using standardized patient scenarios. The results demonstrated a substantial level of agreement, indicating that LLMs can deliver guideline-consistent, literature-informed suggestions. Notably, observed discrepancies emphasize that AI should function as a complementary decision-support tool rather than a substitute for clinical expertise^12^. Given its current inability to interpret imaging, laboratory data, and physical examination findings, AI applications must remain within the framework of individualized, patient-centered care.Hysterectomy is among the most common gynecological procedures worldwide, indicated for conditions such as abnormal uterine bleeding (AUB), fibroids, endometriosis, and pelvic organ prolapse^13^. AUB affects a substantial proportion of women of reproductive age and is a leading benign indication for hysterectomy. While hysterectomy effectively resolves bleeding and can improve quality of life, uterus-sparing approaches such as hysteroscopic management may offer comparable symptom control with fewer complications for selected patients. In the United States, the overall prevalence of hysterectomy is approximately 21% and has remained relatively stable between 2006 and 2016, with higher rates observed in women aged 40 years and older^14–16^. The lifetime risk of hysterectomy is estimated at 45%, with most procedures performed between ages 20 and 49^13^. Evidence from randomized controlled trials indicates that, while hysterectomy effectively eliminates bleeding and can improve quality of life, less invasive alternatives such as the LNG-IUS or conservative surgery may offer comparable quality-of-life benefits with fewer complications for many women^17^. The mean age in our cohort was 48.7 years, consistent with the increased surgical rates seen during the perimenopausal period. The most frequent presenting symptoms were irregular menstruation, heavy menstrual bleeding, and postmenopausal bleeding. Nearly half of the patients had received prior medical therapy, indicating persistent or recurrent symptoms despite conservative management. Leiomyomas were the most common pathological finding in our cohort (70.1%), consistent with epidemiological estimates reporting a cumulative incidence of 70–80% by age 50^18^. Evidence-based management strategies include medical therapy, myomectomy, uterine artery embolization, and hysterectomy, with the choice guided by symptom severity, reproductive preferences, and comorbidities^19^. While ChatGPT-4 frequently recommended hysterectomy for fibroid-related cases, it also suggested uterus-preserving alternatives when clinically appropriate, reflecting an ability to align with individualized care principles.

This study has limitations. AI models do not account for psychosocial, cultural, and emotional influences on treatment choices. The single-center design and modest sample size limit generalizability. Moreover, continuous algorithm refinement and adherence to ethical and data-protection standards are critical for safe clinical integration.

## Conclusion

In this cohort of 87 patients scheduled for hysterectomy, ChatGPT-4 produced recommendations that were largely concordant with physician decisions and occasionally offered reasonable alternatives. The use of a standardized patient-scenario template ensured reproducible, unbiased AI input. Discrepancies were most often linked to fibroid characteristics, symptom burden, anemia, and reproductive goals.

LLMs may enhance decision-making in gynecologic surgery by providing structured, evidence-based options. However, they currently lack integration with physical examination, imaging, laboratory data, and the nuanced understanding of individual patient contexts. Until validated through large, multicenter studies, AI recommendations should be interpreted as supportive input and confirmed by the treating clinician.

## Supplementary Information

Below is the link to the electronic supplementary material.

Supplementary Material 1

## Data Availability

The data generated and/or analyzed during this study are available from the corresponding authors upon reasonable request. The datasets are stored at the Department of Obstetrics and Gynecology, Akdeniz University. For data access, please contact Prof. Dr. M. İlkin Yeral (ilkinaryeraldr@hotmail.com) or Dr. Saltuk Buğra Arıkan (dr_saltukbugra@hotmail.com).
