# Supplementary material for: Comparison of clinical hysterectomy indications with ai-based recommendations: a prospective study
Source: Sci Rep. 2025 Oct 9;15:35385. doi: 10.1038/s41598-025-19216-y (PMC12511411; doi:10.1038/s41598-025-19216-y)
Supplement: Supplementary file 1 — Supplementary Material 1 [file 41598_2025_19216_MOESM1_ESM.pdf]

EXAMPLE PATİENT PROMPTS

PATIENT 18:

Hello ChatGPT, I will now provide information about a patient in our clinic who is scheduled for hysterectomy. Based on this information, I would like you to choose the most medically appropriate treatment option and explain your reasoning in a single sentence enclosed in quotation marks.

Patient age: 50

Gravida: 4

Parite: 4

Delivery method: Vaginal

Menopausal status: No

Symptoms: HMB

Comorbidities: None

History of breast cancer: No

Tamoxifen use: No

Family history of cancer: No

If Yes, Type of cancer: -

Current medications: None

Medical treatment history for current complaint: Yes

Treatment received: Hormonal therapy

Previous surgeries: None

If Yes, Type of surgery:

History of therapeutic hysteroscopy: No

Smoking: No

TVUS endometrial findings: No Endometrıal Thickening

Presence of fibroids on TVUS: Yes

FIGO fibroid Type: Type 6

Fibroid size on TVUS: Larger than 10 Cm

Presence of polyps on TVUS: No

Hemoglobin deficiency: Yes

Endometrial biopsy result: Normal

Surgery planned: Abdominal Hysterectomy

Among the following options:

1. Hysterectomy
2. Myomectomy
3. Hysteroscopy
4. Medical treatment
5. Hormonal IUD (LNG-IUD)
6. Expectant management
7. Hysteroscopy followed by hysterectomy

Please choose the most appropriate option based on this information and write one sentence with your reasoning enclosed in quotation marks.

ChatGPT Response:

Option: "1) Hysterectomy"

Reason: "Considering the patient is 50 years old, presents with heavy menstrual bleeding (HMB), has a fibroid larger than 10 cm, and suffers from anemia, abdominal hysterectomy is considered the most appropriate treatment option."

PATIENT 21:

Hello ChatGPT, I would like to share information about a patient currently scheduled for hysterectomy in our clinic. Based on the following details, please choose the most appropriate medical option and provide a one-sentence explanation for your choice within quotation marks.

Patient age: 45

Gravida: 4

Parite: 4

Delivery method: Vaginal

Menopausal status: No

Symptoms: Irregular Menstruatıon

Comorbidities: None

History of breast cancer: No

Tamoxifen use: No

Family history of cancer: No

Currently used medications: None

Medical treatment history for current complaint: No

Past surgical history: None

Previous hysteroscopic intervention: No

Smoking: No

TVUS findings:

Endometrial findings: No Endometrıal Thickening

Presence of fibroid: Yes

Fibroid Type: FIGO Type 4

Fibroid size: 3-5 cm

Polyp: No

Hemoglobin deficiency: No

Endometrial biopsy result: Normal

Planned surgery: Laparoscopıc Hysterectomy

Among the following options:

1. Hysterectomy
2. Myomectomy
3. Hysteroscopy
4. Medical treatment
5. Hormonal IUD (LNG-IUD)
6. Expectant management
7. Hysteroscopy followed by hysterectomy

Please choose the most suitable treatment option and write one sentence explaining your reasoning between quotation marks."

ChatGPT Response:

Option: “2) Myomectomy”

Rationale: “Considering that the 45-year-old patient has irregular menstruation, a 3-5 cm fibroid, and a normal endometrial biopsy result, myomectomy is considered an effective treatment method that preserves the uterus while addressing the symptoms.”

PATIENT20:

Hello ChatGPT, I will now share information about a patient in our clinic who is currently scheduled for a hysterectomy, and based on this information, I would like you to choose the most appropriate medical option and explain your reasoning in one sentence, enclosed in quotation marks.

Patient age: 45

Gravida: 4

Parite: 4

Delivery method: Vaginal

Menopausal status: No

Symptoms: Irregular Bleedıng

Comorbidities: Hypertensıon

History of breast cancer: No

Tamoxifen use: No

Family history of cancer: No

Current medications: None

Previous medical treatment for current complaint: Yes

Type of treatment: combıned oral contraceptıves

Past surgical history: No

If Yes, Type of surgery:

Previous hysteroscopy: Yes

Smoking status: No

Transvaginal Ultrasound (TVUS) findings: normal

Endometrium: No endometrıal thıckenıng

Fibroid presence: Yes

Fibroid Type: Type 5

Fibroid size: Multıple ıntramural fıbroıds

Polyp presence: No

Hemoglobin deficiency:Yes

Endometrial biopsy result: Normal

Planned surgery: Abdomınal Hysterectomy

For this patient history, please choose the most appropriate among the following treatment options and justify your reasoning in one sentence enclosed in quotation marks:

1. Hysterectomy
2. Myomectomy
3. Hysteroscopy
4. Medical treatment
5. Hormonal IUD (LNG-IUD)
6. Follow-up
7. Hysteroscopy followed by hysterectomy

ChatGPT Response

Option: “1) Hysterectomy”

Rationale: “Considering that the 55-year-old patient has abnormal uterine bleeding, multiple intramural fibroids, and hemoglobin deficiency, abdominal hysterectomy is evaluated as the most appropriate treatment method.”
